# Supplementary material for: Understanding surgical smoke in laparoscopy through Lagrangian Coherent Structures
Source: PLoS One. 2023 Nov 14;18(11):e0293287. doi: 10.1371/journal.pone.0293287 (PMC10645321; doi:10.1371/journal.pone.0293287)
Supplement: S1 Appendix — (PDF) [file pone.0293287.s001.pdf]

S1 Appendix. The equations for the CFD model.
By writing the fluid velocity and the position coordinates in tensor form as u_j and x_j, respectively, with direction j, the continuity equation is given by

$$\frac{\partial u_j}{\partial x_j} = 0. \tag{1}$$

Similarly, the momentum equation can be written as

$$\frac{\partial}{\partial t}(\rho u_i) + \frac{\partial}{\partial x_j}(\rho u_i u_j) = -\frac{\partial p}{\partial x_i} + \frac{\partial \tau_{ij}}{\partial x_j}, \tag{2}$$

where ρ is the fluid density, p, static pressure and the shear stress τ_{ij}, i.e., j^{th} component of the stress acting on the faces of the fluid element perpendicular to axis i, is dependent on the fluid viscosity μ by

$$\tau_{ij} = \mu \left(\frac{\partial u_i}{\partial x_j} + \frac{\partial u_j}{\partial x_i} \right). \tag{3}$$

On the other hand, the energy equations are derived using the $k - \omega$ SST turbulence model which combines the best of the $k - \omega$ model and $k - \omega$ model with a high Reynolds number. Hence, the turbulent kinetic energy k and dissipation rate ω are modelled by different equations where the transport equation for k is

$$\frac{\partial(\rho k)}{\partial t} + \frac{\partial(\rho u_i k)}{\partial x_i} = \frac{\partial}{\partial t}\left((\mu + \sigma_k \mu_t) \frac{\partial k}{\partial x_i} \right) + \tilde{P}_k - \beta^\star \rho \omega k, \tag{4}$$

where the left-hand side corresponds to the time derivative and convection terms for k and the right-hand side has the diffusion, production and dissipation terms, respectively. In particular, a production limiter is employed to avoid the build-up of turbulence in the stagnation regions with

$$P_k = \mu_t \frac{\partial u_i}{\partial x_j} \left(\frac{\partial u_i}{\partial x_j} + \frac{\partial u_j}{\partial x_i} \right), \; \tilde{P}_k = \min(P_k, 10 \cdot \beta^* \rho k \omega).$$

Similarly, the transport equation for ω is

$$\frac{\partial(\rho \omega)}{\partial t} + \frac{\partial(\rho u_i \omega)}{\partial x_i} = \frac{\partial}{\partial x_i}\left((\mu + \mu_t \sigma_\omega) \frac{\partial \omega}{\partial x_i} \right) + \alpha \rho S^2 - \beta \rho \omega^2 + 2(1 - F_1) \frac{\rho \sigma_{\omega_2}}{\omega} \frac{\partial k}{\partial x_j} \frac{\partial \omega}{\partial x_j}, \tag{5}$$

where the blending function

$$F_1 = \tanh\left(\left(\min\left(\max\left(\frac{\sqrt{k}}{\beta^\star \omega y}, \frac{500\nu}{y^2 \omega} \right), \frac{4 \rho \sigma_{\omega_2} k}{CD_{k\omega} y^2} \right) \right)^4 \right),$$

takes a value of 1 at the near wall region to activate the original equation for ω , and gradually switches to 0 moving away from the surface to activate the transformed $k - \epsilon$ equation. Here, y denotes the distance to the nearest surface and

$$CD_{k\omega} = \max\left(\frac{2 \rho \sigma_{\omega_2}}{\omega} \frac{\partial k}{\partial x_j} \frac{\partial \omega}{\partial x_j}, 10^{-10} \right).$$

The turbulent eddy viscosity is defined as

$$\nu_t = \frac{a_1 k}{\max(a_1 \omega, S F_2)}, \tag{6}$$

where S denotes invariant measure of strain rate and F_2 is the second blending function that determines the value of ν_t to be taken and is given by

$$F_2 = \tanh\left(\left(\max\left(2\frac{\sqrt{k}}{\beta^\star \omega y}, \frac{500\nu}{y^2\omega}\right)\right)^2\right).$$

The values of the constants are $\beta^\star = 0.09$, $\alpha_1 = 5/9$, $\beta_1 = 3/40$, $\sigma_{k_1} = 0.85$, $\sigma_{\omega_1} = 0.5$, $\alpha_2 = 0.44$, $\beta_2 = 0.0828$, $\sigma_{k_2} = 1$, $\sigma_{\omega_2} = 0.856$.
